# Small for Gestational Age Calves: Part II—Reduced Fertility, Productive Performance, and Survival in Holstein Friesian Heifers Born Small for Their Gestational Age

**DOI:** 10.3390/ani14152157

**Published:** 2024-07-24

**Authors:** Maya Meesters, Mieke Van Eetvelde, Karel Verdru, Jan Govaere, Geert Opsomer

**Affiliations:** Department of Internal Medicine, Reproduction and Population Medicine, Faculty of Veterinary Medicine, Ghent University, Salisburylaan 133, 9820 Merelbeke, Belgium

**Keywords:** small for gestational age, survival, productive life, fertility

## Abstract

**Simple Summary:**

Recently: more attention has been given to low-birth-weight calves, often without considering gestation length. Similar to human medicine, calves classified as small for gestational age (SGA) weigh below the 10th percentile at birth. The present study aimed to investigate the long-term effects on fertility, productive performance, and overall survival in Holstein Friesian (HF) heifers born SGA. We found that while SGA calves showed initial catch-up growth at six months of age, they were still significantly lighter at twelve months. SGA heifers required significantly more inseminations to conceive compared to their average (AGA) and large (LGA) counterparts. Additionally, more SGA heifers were culled during their first lactation and had lower survival rates to the second calving. SGA heifers also had a higher risk of leaving the herd prematurely. Although overall lifespan and total milk yield were similar among all groups, SGA heifers produced less milk per productive day. This study reveals that HF calves born SGA face significant long-term health and productivity challenges, emphasizing the need for further research on the economic impact of rearing SGA heifers.

**Abstract:**

Recently: more attention has been given to low-birth-weight calves, often without considering gestation length. Calves can be classified as small for gestational age (SGA) when their birth weight is below the 10th percentile, similar to the definition in human medicine. While SGA babies face various health risks, it remains unclear if SGA calves face similar long-term health consequences. This study aimed to investigate the long-term effects on fertility, productive performance, and overall survival in Holstein Friesian (HF) heifers born SGA. Chi-squared analysis assessed culling and survival rates, and linear mixed-effect models evaluated the impact of gestational age group (small, average, or large for gestational age, respectively, SGA, AGA, and LGA) on growth, fertility, milk yield, and lifespan. SGA calves showed catch-up growth at six months but weighed significantly less at twelve months (*p* = 0.003). Age at first insemination and calving did not differ significantly, although SGA heifers required more inseminations (2.3 ± 1.50) compared to AGA and LGA heifers (1.7 ± 0.98 and 1.5 ± 0.89, respectively, *p* = 0.006). SGA calves tended to be culled more during the first lactation than AGA calves (25.0% vs. 11.9%, *p* = 0.078) and showed lower survival to second calving (*p* = 0.019) compared to AGA and LGA heifers. The Kaplan–Meier analysis indicated a tendency for gestational age to affect overall survival (*p* = 0.1), with SGA heifers having a higher risk of leaving the herd prematurely (*p* = 0.035, hazard ratio = 1.53). Milk yield per productive day was significantly lower in SGA heifers (21.2 ± 8.73 kg) compared to AGA and LGA heifers (26.9 ± 5.01 kg and 26.3 ± 3.38 kg, respectively, *p* = 0.006). This study reveals that HF calves born SGA suffer long-term consequences, although further research is needed to understand the economic impact of rearing SGA heifers.

## 1. Introduction

Low birth weights have been acknowledged as a negative prognostic factor for neonatal survival in different mammalian species, like cattle. However, despite its link to a range of health outcomes, the definition of low birth weight is not standardized and even lacking in many breeds of some species [1]. Berglund et al. [2] stated that Swedish Holstein calves with a lower birth weight had a higher incidence of unexplained stillbirth, even though they were born full-term and were clinically normal. More recently, low-birth-weight calves have been described to have higher morbidity [3,4] and mortality risks in the pre-weaning period [3]. Moreover, it has been established that a calf’s birth weight has a significant effect on its predicted 400-day body weight [5]. Consequently, calf birth weight has been described to influence weight at parturition, as well as the interval from parturition to first insemination and milk yield during the first lactation [6]. 

In human medicine, fetal growth abnormalities are often diagnosed using criteria such as low birth weight, macrosomia, and small (SGA) and large for gestational age (LGA) [7]. SGA is most commonly used to describe a newborn with a birth weight below the 10th percentile for gestational age, and its definition can only be applied after birth [8]. It is well known that, in humans, SGA is associated with short-term complications like perinatal morbidity and mortality [9], as well as long-term cardiovascular and metabolic risks [10]. Furthermore, the vast majority of SGA babies will show vigorous catch-up growth in the first two years of life, with an increased propensity to gain intra-abdominal fat, accompanied by a shift from insulin sensitivity to insulin resistance [11,12]. This insulin resistance could contribute to further excess central adiposity [11] and to an early onset of and a rapid progression through puberty in some SGA children [13]. Girls may also develop amplified adrenarche (puberty of the adrenal gland) with or without precocious pubarche (appearance of pubic hair) and early menarche, which can be followed by hyperinsulinemic hyperandrogenism in adolescence, which is a well-known pathway to polycystic ovarian syndrome [13,14].

Recently, more attention has been given to low-birth-weight calves [3,4], although, to the best of our knowledge, there is no available literature considering the birth weight of calves in relation to their gestational age. Therefore, we defined the concept of SGA in Holstein Friesian (HF) calves similar to how it has been characterized in human medicine, and found it applicable to these calves [15]. While it is clear that SGA babies are more at risk to suffer from both short- [9] as well as long-term health risks [10], it remains unknown whether SGA calves suffer similar long-term health consequences, ultimately influencing a dairy cow’s survival and productive lifespan and eventual life-time milk yield.

Thus, the aim of this study was to investigate whether HF calves born SGA are more at risk to suffer from long-term implications concerning reproductive performance, productive lifespan, and overall survival, compared to their average for gestational age (AGA) counterparts. We hypothesized that SGA calves have lower fertility as nulliparous heifers, lower survival, and a lower productive performance compared to their AGA counterparts.

## 2. Materials and Methods

All experimental procedures were approved by the Ethical Committee (EC) of the Faculty of Veterinary Medicine, Ghent University (Belgium), under the EC number 2017/87, were in accordance with the relevant guidelines and regulations, and were in compliance with the ARRIVE guidelines [16].

### 2.1. Farms, Animals, and Management

Data were collected from four dairy farms in Flanders (Belgium). The size of the herds ranged from 100 to 250 lactating cows, with an average 305-day milk yield of 10,000 kg. Three of these herds milked their cows conventionally, twice a day. The fourth herd used an automated milking system that recorded an average of 2.6 milkings a day. All herds participated in official milk recording and provided us their informed consent to access milk recording data. Both nulliparous heifers and cows were housed in free-stall barns.

Based on the monthly milk recordings, animals were fed to their requirements for maintenance and production. Rations consisted of highly qualitative roughages, such as maize and grass silage, sugar beet pulp, and fodder beets, supplemented with concentrates.

The reproductive management of the herds was similar, in that age at first insemination in heifers was between 13 and 15 months, and cows were inseminated at the first observed estrus after a 50-day waiting period. Dry period length averaged six to eight weeks, and animals were separated in a maternity pen when they approached their parturition date. Calves were immediately moved to individual calf pens after parturition and were fed two liters of colostrum. All calves were fed four liters of colostrum within 12 h of birth.

### 2.2. Measurements and Data Collection

A total of 273 female, newborn, and purebred HF calves was enrolled in the study between August 2017 and November 2018. Inclusion criteria consisted of a gestation length between 265 and 295 days, neonatal calf measurements performed within the first ten days after birth, and no missing data. Calf information included in this study was body weight (Seca^®^ flat scale, Seca Benelux, Naarden, The Netherlands) and age at measurement. Dam information included dam parity, classified as nulliparous or multiparous. All calves were classified as SGA, AGA, or LGA based on the optimized birth-weight curves, accounting for dam parity and calf sex. A complete description of the methods used to define SGA, AGA, and LGA calves can be found in Meesters et al. [15]. In short, calves with a real weight below the 10th percentile or above the 90th percentile were classified as SGA and LGA, respectively. Calves with a weight between the 10th and 90th percentile were classified as AGA.

To assess the heifers’ average daily weight gain (ADG) between 0–6 months and 6–12 months, heart girth (HG) was measured to estimate their body weight (EBW) at 6 and at 12 months, using the equation developed by Heinrichs et al. [17], which was recently revalidated for its use [18]: BW (kg) = 65.36 − (1.966 × HG) + (0.01959 × HG^2^) + (0.00001691 × HG^3^).

To determine the body condition of the animals, backfat thickness (BFT) was measured using ultrasonography (Tringa Linear, Esaote/Pie Medical, Maastricht, The Netherlands), to objectively and accurately assess fat reserves in a non-invasive way, as described by Schröder and Staufenbiel [19]. The heifers’ BFT was repeatedly measured, with key measuring moments at 6 months and around the first artificial insemination. 

To elucidate the reproductive performance and productive lifespan of the 273 heifers enrolled, all animals were followed until they left the farm, with 15 July 2023 as an absolute end date of data collection. Data were collected with informed consent by the farmers, using the farm’s management program (UNIFORM-Agri^®^, Stationsplein 14, 9401 LB Assen, The Netherlands). Fertility data comprised of age at first insemination, the number of artificial inseminations (AIs) until pregnancy, and age at conception. Also, ages at first, second, third, and further parturitions were included. 

Production data comprised the 305-day (305d) milk, fat, and protein yields per lactation, as well as each heifer’s total milk production during their productive life on the farm. In addition, the first lactation 305-day energy-corrected milk yield (305d ECM) was estimated based on 305d milk, fat, and protein yields [20], and was calculated as follows: ECM = [0.3246 × 305d milk production (kg)] + [12.86 × 305d fat production (kg)] + [7.04 × 305d protein production (kg)].

To investigate the heifers’ lifetime performance, lifespan, productive life (including dry-off), milk yield per day of life, and milk yield per productive day were calculated using the following definitions: ‘Lifespan (d)’ = Culling or Death date/Birth date; ‘Productive life (d)’ = Culling or Death date/Date of first calving; ‘Milk per day of life (kg)’ = Lifetime milk yield/Lifespan; ‘Milk per productive day (kg)’ = Lifetime milk yield/Productive life.

Lastly, survival of each animal was calculated using the date of and reason for culling or death. Categories were used to examine when, during their lifetime, death or culling occurred, namely at birth, at 0–6 months, 6–12 months, between 12 months and first AI, between conception and first parturition, and during each recorded lactation. Reasons for culling were also recorded: being ‘dead’ or ‘sold’. Within the ‘sold’ group, reasons for selling were recorded as ‘lameness’, ‘fertility’, ‘disease’, ‘production’, and ‘udder’. As such, fertility as nulliparous animal, survival, milk yields, and productive lifespan for each gestational age category (SGA, AGA, and LGA) could be calculated. 

### 2.3. Statistical Analysis

All statistical analyses were performed using R 3.6.1 [21].

Categorical data, such as culling by or survival until a specific time point (1 = yes/0 = no), were compared between SGA, AGA, and LGA heifers using a chi-squared analysis (Chisq.test() function).

A survival analysis was performed on all animals. A censoring variable was used to differentiate animals that had been sold or died (censor = 1) from the animals that were alive at the end of the data collection (censor = 0). Survival data were analyzed by Kaplan–Meier survival analysis, using the Survival package in R [22]. For further comparisons between SGA, AGA, and LGA heifers, Cox regression was performed using the coxph() function in the same package.

Continuous data on body growth, fertility, milk production, and lifespan were compared between groups with linear mixed-effect models, using the lmer() function of the ‘lme4’ package [23]. The growth, fertility, production, and lifespan variables were the outcomes of the models, whereas gestational age group (SGA, AGA, or LGA) was the explaining fixed effect. Herd was included as a random effect in all models. 

Statistical significance and tendency were declared at *p* < 0.05 and 0.05 < *p* ≤ 0.1, respectively.

## 3. Results

### 3.1. Survival and Culling

The proportion of SGA, AGA, and LGA calves reaching a first AI, conception, and a first until a fifth calving can be found in Table 1.

The proportion SGA calves reaching a first AI was 79.5%, which tended to be lower than in AGA calves (89.4%, *p* = 0.081). In addition, the proportion of calves reaching conception tended to be smaller in SGA versus AGA calves (72.7% and 85.5%, respectively, *p* = 0.062). The proportion of calves reaching their first calving did not differ significantly between the three gestational age groups, although a significantly lower proportion of SGA calves (54.5%) survived until a second calving compared to the AGA (73.9%) and LGA (81.9%) calves (*p* = 0.019). 

At the end of our data collection, 116 out of the 273 (42.5%) cows from our study were still in their second (1 SGA), third (3 SGA, 51 AGA, and 6 LGA), fourth (9 SGA, 39 AGA, and 4 LGA), or fifth (3 AGA) ongoing lactations. At this timepoint, 29.5% of SGA calves were still alive, compared to 44.9% and 45.5% of the AGA and LGA calves, respectively. One SGA cow was still in her second lactation due to a calving interval of 632 days between her first and second parturitions; as such, no statistical analysis was performed for survival until a third calving.

Table 2 shows the distribution of when, during their lifetime, SGA, AGA, and LGA calves were eventually culled.

Calves that died between birth and the age of six months all died because of disease. The proportion of SGA calves that died before the age of six months (11.4%) was numerically higher than that of AGA (7.2%) and LGA (4.5%) calves, albeit not significantly. Calves dying between the ages of six and twelve months died because of disease or an accident. Similarly, a numerical but non-significant difference could be observed, with a greater loss of SGA (5.1%) compared to AGA (1.0%) and LGA (0.0%) calves. The proportions of SGA, AGA, and LGA calves being sold between twelve months of age and first AI, as well as due to the inability to conceive (going from one to nine AIs), did not differ significantly between groups, although numerically, SGAs seemed to be culled more during these timepoints.

In total, 82.4% (225/273) of the heifers in our study reached at least a first calving. The proportion of SGA calves being culled during their first lactation (25.0%) tended to be larger compared to AGA calves (11.9%, *p* = 0.078). No such tendency nor significance could be seen in the second lactation, and as the third through fifth lactations are still ongoing, no further analyses could be performed. Of the eight SGA calves that did not reach a second calving, three died between 12 and 20 days post-parturition due to disease, three were sold because they did not become pregnant, and two more were sold because of underwhelming production and disease. The average age of the SGAs that were culled during their first lactation was 1181 ± 459.0 days (745–1265 days).

**Figure 1:**
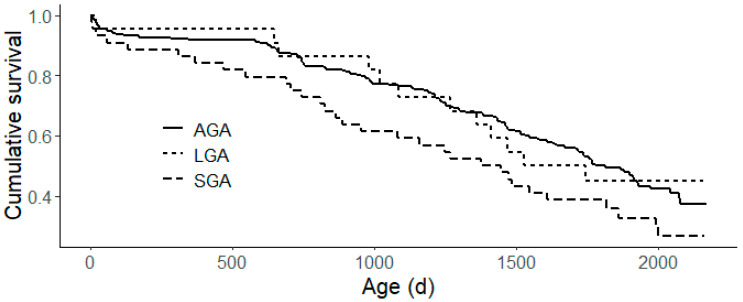
Kaplan–Meier survival analysis plot of survival for each gestational age group. Small for gestational age (SGA) calves have a significantly higher risk (*p* = 0.035, hazard ratio = 1.53) of leaving the herd, compared to their average (AGA) and large for gestational age (LGA) counterparts.

Survival analysis revealed a median survival age of 1745 days (95% CI: 1572–1913) in the studied population. The Kaplan–Meier survival analysis (Figure 1) revealed a tendency for the gestational age group to affect overall survival (*p* = 0.1). The Cox regression analysis showed a similar survival for LGA and AGA heifers in the herd (*p* > 0.9). However, SGA heifers have a significantly higher risk of leaving the herd compared to their AGA and LGA counterparts (*p* = 0.035, hazard ratio = 1.53). 

By the end date of our data collection (15 July 2023), a total of 48.4% (109/225) of the animals that reached a first lactation was culled. Of these animals, 24.8% (27/109) died and 75.2% (82/109) were sold. From these 27 animals, 16 had a recorded reason of death, with most of the animals (11/16) dying from ‘disease’, other than ‘fertility’ (3/16), ‘accident’ (1/16), or ‘udder’ (1/16) issues. Reasons for selling the animals were recorded for 67 out of 82 animals (81.7%). The most common reason for selling a lactating animal was ‘fertility’, with 41.8% (28/67), followed by 22.4% (15/67) due to ‘lameness’, 19.4% (13/67) due to ‘udder’ problems, 10.4% (7/67) because of ‘disease’, and 6.0% (4/67) because of underwhelming ‘production’. 

### 3.2. Growth and Body Conditions in the First Year of Life

There is no significant difference in body weight or average daily gain in the first six months of life between the gestational age groups (Table 3). Yet, SGA calves weighed significantly less at twelve months of age (330 ± 31.3 kg, *p* = 0.003) and tended to grow less (799 ± 145.2 g/d, *p* = 0.10) between six and twelve months of age compared to the AGA and LGA calves. 

The backfat thickness around first AI was on average 11.3 ± 2.86 mm and did not differ significantly between gestational age groups (*p* = 0.54).

### 3.3. Fertility

The fertility parameters until first calving are depicted in Table 4. 

The average age at first insemination was 425 ± 56.9 days and did not differ significantly between gestational age groups. The number of AIs per inseminated heifer and number of AIs per pregnancy were on average 1.8 ± 1.22 (ranging from 1 to 9 AIs) and 1.7 ± 1.08 (ranging from 1 to 6 AIs), respectively. SGA heifers needed 2.3 ± 1.50 AIs to conceive, which was significantly more than their AGA and LGA counterparts (1.7 ± 0.98 and 1.5 ± 0.89, respectively, *p* = 0.006). 

Age at conception was on average 449 ± 69.8 days and did not significantly differ between gestational age groups. Similarly, age at first calving was not significantly different between groups, averaging 723 ± 71.8 days.

### 3.4. Milk Production and Productive Lifespan

During first lactation, lactation length, 305 d milk, fat and protein yields, as well as 305 d ECM were not significantly different between the SGA, AGA, and LGA heifers (Table 5).

The lifetime performance of all animals that were culled or had died by 15 July 2023 is depicted in Table 6. The mean lifespan was 1087 ± 585.0 days, the productive life was 678 ± 358.6 days, and the lifetime milk yield was on average 19,019 ± 10,861.9 kg. There was no significant difference between the different gestational age groups in these variables. Milk yield per day of life, which averaged at 12.4 ± 5.21 kg, also did not differ between SGA, AGA, and LGA calves (*p* = 0.11), although in the multiple comparisons, SGA heifers tended to have a lower average milk yield per day of life than AGA calves (*p* = 0.096). SGA heifers produced significantly less milk per productive day of life (*p* = 0.006) than their AGA and LGA counterparts. Regarding the multiple comparisons, SGA heifers still produced significantly less milk per productive day (*p* = 0.004) when compared to the AGA heifers.

## 4. Discussion

The present study investigated the consequences of being born SGA, in Holstein Friesian dairy cattle. We hypothesized that SGA calves would have a lower survival, a lower fertility as nulliparous heifers, and a lower productive lifespan compared to their AGA counterparts. To the best of our knowledge, this is the first study assessing the long-term outcomes of Holstein Friesian calves born small for their gestational age.

On average, calf mortality between birth and six months was 7.7% for all calves. This is higher than the average rate of 3.4% reported by Wathes et al. [24] covering calf mortality from the age of one to six months. In our study, neonatal mortality, between 24 h and 28 days, was not investigated separately, which could account for the higher mortality rates, as neonatal mortality can range from 0% to 12%, depending on the farm [24,25]. Although no significant difference of calf mortality between birth and six months of age was apparent between the gestational age groups, SGA calves experienced numerically higher death rates (11.4%) compared to their AGA (7.2%) and LGA (4.5%) counterparts. A limitation of this study is that we did not assess perinatal morbidity and mortality in the first 24 to 48 h of life, as calves in our study were weighed and measured within ten days after birth, and as such, we might have missed some SGA calves or have missed calves that died before we could include them in this study. Perinatal morbidity and mortality have been described to be significantly increased in infants born below the 10th percentile for their gestational age [9], and in Swedish Holstein calves, there was a higher rate of unexplained death in lower compared to average birth weight calves [2]. Similar to calf mortality between birth and six months, juvenile mortality between six and fifteen months did not differ between the gestational age groups and was on average 2.6%. This is comparable to the 2.6% to 3.5% juvenile mortality described by Wathes et al. [24]. However, survival until first insemination and until conception tended to be lower in SGA compared to AGA calves. This tendency might be explained by the numerically higher proportion of calf and juvenile mortality in SGA compared to AGA calves, as well as the numerically higher proportion of calves sold due to the inability to conceive. The non-significance in survival until first insemination and until conception between SGA and LGA calves is mainly due to the low numbers in the LGA group, therefore lacking power to achieve statistical significance. All of the SGAs that conceived (72.2%), calved and started a first lactation. However, culling during the first lactation tended to be higher for the SGA compared to the AGA calves, with 25% of the SGAs starting a first lactation being culled during this lactation. The two main reasons for culling in the SGA group were death due to disease (3/8) and sold due to infertility reasons (3/8). There was no significant difference in the culling rate during first lactation between the SGA and LGA calves, mainly due to low numbers in the LGA group. Nevertheless, survival until a second calving was significantly lower in SGA calves compared to their AGA and LGA counterparts: only 54.5% of the SGA calves reached a second calving, compared to 73.9% of the AGA and 81.9% of the LGA calves (*p* = 0.019). It seems that SGA calves are more likely to be culled before reaching a second calving and have a significantly higher risk of leaving the herd prematurely compared to AGA and LGA calves. Even though most deaths in SGA humans occurred during the first year of life, the cumulative mortality risk has been shown to increase until the age of 30 years [26]. This might be similar for SGA calves, with a cumulative increased mortality risk over time. As cows calve for the first time at two years of age, and with an average productive lifespan between 2.5 and 4 years, the average total lifespan from birth to death in dairy cows is between 4.5 to 6 years [27]. With almost half of the SGA heifers not surviving until a second calving (on average 3.2 years old), the average total lifespan of these culled SGA heifers does not reach the average described by De Vries and Marcondes [27], which would be a concern on a dairy farm from both an animal well-being and economical perspective, as cows only become profitable after three lactations [28,29,30]. However, further research on larger study populations is needed to assess overall mortality or culling rates in SGA calves as well as the economic implications for the dairy industry.

The estimated body weight at six months did not differ between the gestational age groups, which might be due to some catch-up growth during the first six months. However, this is not reflected in the average daily weight gain of the SGA calves, as it was numerically higher but did not differ significantly from that of the AGA and LGA calves. Swali and Wathes [31] described that the low-birth-weight offspring of primiparous dairy cows experienced catch-up growth in the first three months of life compared to low-birth-weight calves born to multiparous dams. This reveals another limitation in our study, as we were not able to calculate the average daily gain in the first three months of life and then from three to six months of life. This might have shown a similar catch-up growth in the pre-weaning period and could explain a similar daily weight gain and body weight by the age of six months. Interestingly, in humans, the vast majority of healthy full-term and singleton SGA infants shows catch-up growth in height during the first six to twelve months of life. If catch-up growth does not occur during this early window, about 50% of SGA individuals will remain short as adults [32]. However, if there was some catch-up growth by the SGA calves before the age of six months, this was not sustained between six and twelve months, as their estimated body weight at twelve months was significantly lower than that of their AGA and LGA counterparts (*p* = 0.003) and they tended to have a lower average daily weight gain between six and twelve months of age (*p* = 0.10). In humans, SGA children without spontaneous catch-up growth may be treated with growth hormone (GH), although there are some long-term consequences, like changes in body composition and insulin resistance [10]. Thus, the somatotropic axis, including GH, insulin, insulin-like growth factors (IGFs), and their binding proteins and receptors, which is a key regulator of growth, reproduction, lactation, and health [33], might exhibit varying degrees of resistance along the GH-IGF-insulin signaling pathway [34] in SGA calves. Greenwood and Bell [35] describe that growth-retarded newborn lambs are characterized by very high concentrations of GH, but low concentrations of IGF-1, indicating the apparent immaturity of the somatotropic axis [36], which persists for several weeks after birth [35]. Subsequently, IGF-1 concentrations increase more in small vs. normal-birth-weight lambs, suggesting the resetting of the somatotropic axis during the postnatal period [35]. As higher IGF-1 concentrations are positively associated with growth rates in calves [37,38], being born SGA may limit their ability for catch-up growth in the early postnatal period, at which time calves are especially sensitive to infection [34]. We anticipate similar results as described by Greenwood and Bell [35], with SGA calves having higher circulating GH, lower IGF-1, and higher levels of plasma insulin compared to their AGA counterparts. This could account for the lack of catch-up growth, lower body weight at twelve months of age, and might reveal a potential predisposition to develop insulin resistance. Unfortunately, in the present study, no blood sampling was performed to investigate the function of the somatotropic axis by measuring GH, IGF-1, and insulin. Additionally, there was no difference in the backfat thickness at the age of first insemination, even though more adiposity was expected in the SGA heifers. Posont and Yates [39] describe an altered body composition in low-birth-weight livestock by redirecting nutrients more as visceral fat deposits compared to normal-birth-weight livestock. This is in line with what is described in humans, where SGA babies have an increased propensity to gain central and intra-abdominal fat [11,12,40]. However, in our study, SGA calves seem to have a similar body composition around first AI as their AGA and LGA counterparts. This is in agreement with a human study where, at the beginning of puberty, SGA-born girls had a similar BMI and body composition to AGA girls, although SGA girls exhibited higher blood leptin levels as well as a higher insulinogenic index, which may be early indicators of an underlying degree of insulin resistance [41]. Previous epidemiological studies by Hales and Barker [42] indicated that low birth weight and SGA were associated with increased risks for insulin resistance and metabolic syndrome. As insulin also plays a pivotal role in the development of metabolic and reproductive disorders in dairy cattle [43,44], further research about catch-up growth, body composition in SGA-born heifers, and blood insulin levels to determine the presence of insulin resistance and metabolic function is warranted. Catch-up growth could be more accurately assessed by increasing the weight and body measurements during the first three months. Body proportions and body composition (e.g., fat distribution) at six months and around first service could be investigated using backfat thickness measurements or photographs, as depicted by D. C. Wathes [34]. Long, et al. [45] revealed that nutritional restriction during gestation impairs pituitary gland development in beef heifer offspring. Their finding may have major implications in the regulation of the compensatory growth seen in heifers born to nutrient-restricted dams, as well as altered glucose regulation and increased adiposity [45]. Additionally, low-birth-weight calves experiencing early catch-up growth might have altered fat distribution and insulin sensitivity, potentially leading to suboptimal future fertility [34,46,47,48]. Changes in the metabolic status together with an impaired development of the pituitary gland might have effects on the hypothalamic–pituitary–gonadal axis, and as such influence future fertility.

Despite the lower estimated body weight at twelve months, age at first insemination was similar between all gestational age groups. However, significantly more AIs were recorded per inseminated heifer (*p* = 0.01) and more AIs until pregnancy (*p* = 0.006) were needed in SGA versus AGA and LGA calves. In human medicine, there is evidence that girls born preterm or with a very-low birth weight have lower reproductive success and are more likely to give birth to preterm infants [49,50], although SGA females do not seem to be more infertile than their AGA counterparts [51]. Nonetheless, prenatal growth restraint may be associated with high levels of FSH (suggesting a reduced granulosa cell reserve) and smaller internal genitalia (uterus and ovaries) in adolescence [52]. In cattle, daughters of nutrient-restricted dams had consistently lower peripheral anti-mullerian hormone (AMH) concentrations, increased FSH concentrations, and a lower antral follicle count (AFC), even though gestation length and birth weight were similar in the restricted and non-restricted groups [53]. This warrants further research on SGA calves, as ruminants are born with a limited ovarian reserve, which declines with age and may influence fertility in cattle [54]. Furthermore, de Zegher and Ibáñez [14] also described a hyperinsulinemic pathway to polycystic ovarian syndrome in girls. Abnormal levels of insulin and IGF-1 have been shown to play a role in the development in cystic ovarian disease in dairy cows [55], and as cystic follicles are an important ovarian dysfunction and a major cause of subfertility in dairy cattle [56], further research on SGA calves and possible subfertility due to ovarian dysfunction is warranted. Not only would blood sampling and assessing IGF-1, insulin, and AMH at birth and around puberty be interesting to compare SGA calves with their average-sized counterparts, it might also be interesting to investigate the size of the uterus and ovaries before and at puberty, as well as around first insemination, to corroborate the findings in humans [52].

First lactation performance did not differ significantly between the gestational age groups, regarding lactation length, 305d milk, fat, protein, and ECM. Although there are some numerical differences, SGA calves’ first lactation milk yield matches that of their AGA and LGA counterparts. These results are in accordance with Swali and Wathes [57], who described no adverse effects of small birth size on productivity in the first lactation. Similarly, the lifetime performance of the animals that were culled by the end of our data collection (15 July 2023) revealed numerical but non-significant differences in lifespan, productive life, and lifetime milk yield. Also, SGA calves’ milk yield per day of life did not differ significantly from that of AGA and LGA calves. However, when looking at multiple comparisons, SGA calves tended (*p* = 0.096) to have a lower average milk yield per day of life compared to the AGA calves. This is not surprising, knowing that SGA calves tend to be culled more frequently during their first lactation and show significantly lower survival until a second calving than their AGA and LGA counterparts. More interesting is that the milk yield per productive day (starting from the first calving, thus not influenced by the long rearing period) is significantly lower (*p* = 0.006) in SGA than AGA and LGA animals. During their productive life, SGA heifers produce more than five kg a day less than their AGA and LGA counterparts. This might be an indication of the fact that SGA heifers never fully reach their potential because of underwhelming milk production or because they are culled prematurely. This significantly lower milk yield per productive day is an economic concern for dairy farms; thus, it is debatable whether or not it is in the farms’ best interests to raise SGA heifers to become lactating dairy cows. It might be more interesting to redirect SGA calves to the veal sector, however whether or not this would be economically feasible and more profitable should be elucidated further.

## 5. Conclusions

This study reveals that HF calves may suffer long-term consequences of being born SGA. SGA heifers do not differ in weight at six months of age, but weighed less at twelve months old, meaning they might fail to maintain similar average daily weight gains than AGA and LGA calves. In addition, the fertility of SGA heifers is impaired as they require more inseminations per conception than their AGA and LGA counterparts. Furthermore, SGA heifers have lower survival until a second calving and show a significantly higher risk of leaving the herd prematurely, compared to AGA and LGA heifers. Also, SGA heifers produce significantly less milk per productive day, which indicates they do not reach their full potential. Whether or not it is economically advantageous to rear SGA calves to become lactating dairy cows requires further research on larger study groups.

## Figures and Tables

**Table 1:** Survival of small, average, and large for gestational age (AGA, AGA, and LGA, respectively) calves until different timepoints in their life.

Survival Until	SGA		AGA		LGA		*p*-Value *
	N	%	N	%	N	%	
Birth	44	100.0%	207	100.0%	22	100.0%	-
First AI	35	79.5%	185	89.4%	21	95.5%	0.081
Conception	32	72.7%	177	85.5%	20	90.9%	0.062
1st Calving	32	72.7%	174	84.1%	19	86.4%	0.176
2nd Calving	24	54.5% ^a^	153	73.9% ^b^	18	81.8% ^b^	0.019
*3rd Calving °*	*17*	*38.6%*	*120*	*58.0%*	*11*	*50.0%*	-
*4th Calving*	*11*		*46*		*4*		-
*5th calving*	*0*		*3*		*0*		-
15 July 2023	13	29.5%	93	44.9%	10	45.5%	

° One SGA still in second lactation. * Different superscripts indicate significant differences between SGA, AGA and LGA calves (*p* < 0.05).

**Table 2:** Distribution of the timings of the culling of small, average, and large for gestational age (SGA, AGA, and LGA, respectively) calves.

	All			SGA			AGA			LGA			*p*-Value
	N	% Tot. (N = 273)	% Rem.	N	% Tot. (N = 44)	% Rem.	N	% Tot. (N = 207)	% Rem.	N	% Tot. (N = 22)	% Rem.	
Birth	0	0.0%	0.0%	0	0.0%	0.0%	0	0.0%	0.0%	0	0.0%	0.0%	
Dead at 0–6 M	21	7.7%	7.7%	5	11.4%	11.4%	15	7.2%	7.2%	1	4.5%	4.5%	0.63
Dead at 6–12 M	4	1.5%	1.6%	2	4.5%	5.1%	2	1.0%	1.0%	0	0.0%	0.0%	0.14
Sold at 12 M–1st AI	7	2.6%	2.8%	2	4.5%	5.4%	5	2.4%	2.6%	0	0.0%	0.0%	0.52
Sold: inability to conceive	12	4.4%	5.0%	3	6.8%	8.6%	8	3.9%	4.3%	1	4.5%	4.8%	0.75
Dead/sold during 1st Lact.	30	11.0%	13.3%	8	18.2%	25.0%	21	10.1%	11.9%	1	4.5%	5.3%	0.077
Dead/sold during 2nd Lact.	46	16.8%	23.5%	6	13.6%	25.0%	33	15.9%	21.2%	7	31.8%	38.9%	0.27
Dead/sold during 3rd Lact.	27	9.9%	18.0%	3	6.8%	16.7%	23	11.1%	18.7%	1	4.5%	9.1%	
Dead/sold during 4th Lact.	6	2.2%	4.9%	2	4.5%	13.3%	4	1.9%	4.0%	0	0.0%	0.0%	

% Tot. = % of total; % Rem. = % of remaining; Lact. = lactation.

**Table 3:** Estimated body weight (EBW), average daily weight gain (ADG), and backfat thickness (BFT) of small, average, and large for gestational age (SGA, AGA, and LGA, respectively) calves in the first year of life.

	ALL		SGA			AGA			LGA			*p*-Value *
	N	Mean ± SD	N	Mean ± SD	IQR	N	Mean ± SD	IQR	N	Mean ± SD	IQR	
EBW 6 M (kg)	250	191 ± 25.9	39	187 ± 25.53	175.7–20.8	190	192 ± 26.6	178.7–208.2	21	198 ± 18.5	181.8–214.2	0.14
EBW 12 M (kg)	232	343 ± 43.0	35	330 ± 31.3 ^a^	316.6–353.7	177	344 ± 44.7 ^b^	323.1–372.5	20	358 ± 40.4 ^b^	331.0–392.0	0.003
ADG 0–6 M (g/d)	250	856 ± 147.1	39	858 ± 148.4	793.0–955.4	190	856 ± 151.8	787.1–955.0	21	856 ± 98.4	777.2–944.2	0.90
ADG 6–12 M (g/d)	231	847 ± 170.3	35	799 ± 145.2	757.8–896.7	176	853 ± 175.9	749.4–965.5	20	887 ± 179.0	833.5–1009.0	0.10
BFT 1st AI (mm)	224	11.3 ± 2.86	34	11.1 ± 2.25	10.3–12.0	170	11.4 ± 2.92	9.0–13.0	20	11.4 ± 3.34	9.0–13.3	0.54

* Different superscripts indicate significant differences between SGA, AGA and LGA calves (*p* < 0.05). IQR: interquartile range.

**Table 4:** Fertility parameters until first calving in SGA, AGA, and LGA heifers.

	All		SGA			AGA			LGA			*p*-Value *
	N	Mean ± SD	N	Mean ± SD	IQR	N	Mean ± SD	IQR	N	Mean ± SD	IQR	
Age 1st AI (d)	241	425 ± 56.9	35	425 ± 46.8	392–454	185	426 ± 58.3	379–458	21	418 ± 61.3	376–453	0.78
# AI/ins. heifer	241	1.8 ± 1.22	35	2.4 ± 1.57 ^a^	2.0–3.0	185	1.7 ± 1.14 ^b^	1.0–2.0	21	1.5 ± 0.87 ^b^	1.0–2.0	0.01
# AI/p	229	1.7 ± 1.08	32	2.3 ± 1.50 ^a^	2.0–3.0	177	1.7 ± 0.98 ^b^	1.0–2.0	20	1.5 ± 0.89 ^b^	1.0–2.0	0.006
Age conception (d)	229	449 ± 69.8	32	469 ± 61.0	419–503	177	448 ± 71.4	396–477	20	424 ± 61.7	381–453	0.22
Age 1st calving (d)	225	723 ± 71.8	32	744 ± 63.6	698–786	174	722 ± 73.2	670–749	19	696 ± 63.4	652–730	0.17

d = days, # AI/ins; heifer = number of AIs per inseminated heifer; # AI/*p* = number of AIs per pregnancy; IQR: interquartile range. * Different superscripts indicate significant differences between SGA, AGA and LGA calves (*p* < 0.05).

**Table 5:** First lactation performance in SGA, AGA, and LGA heifers.

	All		SGA			AGA			LGA			*p*-Value
	N	Mean ± SD	N	Mean ± SD	IQR	N	Mean ± SD	IQR	N	Mean ± SD	IQR	
Lactation length (d)	222	324 ± 103.1	31	296 ± 116.7	286–331	172	328 ± 104.4	285–346	19	333 ± 50.5	310–345	0.26
305d milk (kg)	222	9189 ± 1615.4	31	8765 ± 1688.7	7319–10175	172	9213 ± 1609.2	8172–10,221	19	9667 ± 1458.6	8550–10,728	0.23
305d fat (kg)	222	374 ± 58.0	31	369 ± 68.7	315–414	172	372 ± 55.9	336–413	19	398 ± 55.6	360–425	0.53
305d protein (kg)	222	315 ± 53.6	31	307 ± 59.5	257–348	172	315 ± 53.1	285–353	19	329 ± 47.8	297–261	0.34
305d ECM (kg)	222	10,005 ± 1562.2	31	9745 ± 1788.3	8423–11,036	172	9990 ± 1525.0	8964–11,065	19	10,569 ± 1443.6	9562–11,490	0.38

IQR: interquartile range.

**Table 6:** Lifetime performance of the culled SGA, AGA, and LGA heifers.

	All		SGA			AGA			LGA			*p*-Value *
	N	Mean ± SD	N	Mean ± SD	IQR	N	Mean ± SD	IQR	N	Mean ± SD	IQR	
Lifespan (d)	157	1087 ± 585.0	31	959 ± 612.1	507–1457	114	1121 ± 587.4	731–1577	12	1099 ± 477.7	902–1425	0.51
Productive life (d)	109	678 ± 358.6	19	597 ± 424.3	234–877	81	702 ± 352.3	456–1008	9	633 ± 256.9	448–817	0.48
Lifetime milk (kg)	83	19,019 ± 10,861.9	13	15,392 ± 13,513.8	2781–25,108	63	20,017 ± 10,419.0	11,220–26,183	7	16,772 ± 8976.3	9202–22,345	0.54
Milk per day of life (kg)	83	12.4 ± 5.21	13	9.5 ± 6.72	3.1–13.8	63	13.0 ± 4.83	9.7–16.2	7	12.3 ± 4.08	8.8–14.9	0.11
Milk per productive day (kg)	83	25.9 ± 5.94	13	21.2 ± 8.73 ^a^	20.0–28.5	63	26.9 ± 5.01 ^b^	24.7–30.4	7	26.3 ± 3.38 ^b^	24.8–28.3	0.006

‘Lifespan’ = culling or death date—birth date; ‘Productive life’ = culling or death date—date of first calving; ‘Milk per day of life’ = Lifetime milk yield/lifespan; ‘Milk per productive day’ = Lifetime milk yield/productive life. IQR: interquartile range. * Different superscripts indicate significant differences between SGA, AGA and LGA calves (*p* < 0.05).

## Data Availability

The datasets presented in this article are not readily available because the data are part of an ongoing study. Requests to access the datasets should be directed to the corresponding authors.
